# Oral contraceptive pill (OCP) treatment alters the gene expression of intercellular adhesion molecule-1 (ICAM-1), tumor necrosis factor-α (TNF-α), monocyte chemoattractant protein-1 (MCP-1) and plasminogen activator inhibitor-1 (PAI-1) in polycystic ovary syndrome (PCOS) women compared to drug-naive PCOS women

**DOI:** 10.1186/s12905-023-02187-5

**Published:** 2023-02-15

**Authors:** Syed Douhath Yousuf, Mohammad Ashraf Ganie, Uneeb Urwat, Syed Mudasir Andrabi, Mohammad Afzal Zargar, Mashooq Ahmad Dar, Mir Manzoor-ul-Rehman, Syed Mudassar, Fouzia Rashid

**Affiliations:** 1 Department of Clinical Biochemistry, Sheri- Kashmir Institute of Medical Sciences, SKIMS, Srinagar, J&K India; 2 Department of Endocrinology and Metabolism, Sheri- Kashmir Institute of Medical Sciences, SKIMS, Srinagar, J&K India; 3 Division of Animal Biotechnology, Sheri- Kashmir Institute of Agricultural Sciences, Shuhama, J&K India; 4 Central University of Kashmir, Ganderbal, J&K India; 5 Division of Animal Biochemistry, Sheri- Kashmir Institute of Agricultural Sciences, Shuhama, J&K India; 6 Clinical Biochemistry, University of Kashmir, Srinagar, J&K India

**Keywords:** Polycystic ovary syndrome, Oral contraceptive pill, Tumor necrosis factor-α, Plasminogen activator inhabitor-1, Monocyte chemoattractant protein-1, Inflammation, Coagulation, Insulin resistance

## Abstract

**Background:**

Polycystic ovary syndrome (PCOS) presents clinical symptoms of menstrual abnormalities, excessive hair growth (hirsutism), scalp hair loss, acne and infertility. Metabolic abnormalities such as obesity, insulin resistance, glucose intolerance and cardiovascular problems constitute an essential part of PCOS, all of which can have significant long-term health consequences. Low-grade chronic inflammation demonstrated by persistent moderately elevated serum levels of inflammatory and coagulatory markers plays a critical role in the pathogenesis of PCOS. Oral contraceptive pills (OCPs) constitute the mainstay of pharmacologic therapy for women with PCOS to regularize cyclicity and ameliorate androgen excess. On the other hand, OCP use is associated with various venous thromboembolic and proinflammatory events in the general population. PCOS women always carriers the increased lifetime risk of these events. The studies on the effect of OCPs on inflammatory, coagulation and metabolic parameters in PCOS are less robust. Therefore in this study, we investigated and compared the messenger RNA (mRNA) expression profiles of genes implicated in inflammatory and coagulation pathways between drug-naive and OCP-treated PCOS women. The selected genes include intercellular adhesion molecule-1 (ICAM-1), tumor necrosis factor-α (TNF-α), monocyte chemoattractant protein-1 (MCP-1) and plasminogen activator inhibitor-1 (PAI-1). Furthermore, the correlation between the selected markers and various metabolic indices in the OCP group has also been explored.

**Method:**

The relative amounts of ICAM-1, TNF-α, MCP-1 and PAI-1 mRNA in peripheral blood mononuclear cells from 25 drug-naive PCOS subjects (controls) and 25 PCOS subjects who received OCPs containing 0.03 mg-ethinyl-estradiol and 0.15 mg-levonorgestrel for at least six months (cases) were estimated using real-time qPCR. The statistical interpretation was conducted using SPSS version 20.0 (SPSS, Inc, Chicago, IL), Epi Info version 2002 (Disease Control and Prevention Centres, Atlanta, GA) and GraphPad Prism 5 (GraphPad Software, La Jolla, CA) software.

**Result:**

Six months of OCP therapy enhanced the expression of inflammatory genes viz ICAM-1, TNF-α and MCP-1 mRNA in PCOS women by 2.54, 2.05 and 1.74 folds, respectively, in this study. However, PAI-1 mRNA in the OCP group showed no significant increase. Furthermore, in cases, ICAM-1 mRNA expression positively correlated with body mass index (BMI) (p = 0.01), fasting insulin (p = 0.01), insulin 2 h p = 0.02), glucose 2 h (p = 0.01) and triglycerides (p = 0.01). TNF-α mRNA expression positively correlated with fasting insulin (p = 0.0007). MCP-1 mRNA expression positively correlated with (BMI) (p = 0.002).

**Conclusion:**

OCPs helped reduce clinical hyperandrogenism and regularise menstrual cycles in women with PCOS. However, OCP use was associated with increased fold expression of inflammatory markers which positively correlated with metabolic abnormalities.

## Background

Polycystic ovary syndrome (PCOS) is one of the most common endocrine diseases, involving 6–20% percent of women of reproductive age [1], with an even greater incidence among Kashmiri women [2]. Apart from the symptoms of hyperandrogenism and infertility, PCOS is associated with several metabolic abnormalities, including obesity, insulin resistance (IR), hyperlipidemia, systemic inflammation and functional atherosclerosis [3–5]. Elevated inflammatory and coagulatory markers characterize this low-grade inflammation and endothelial dysfunction. Intercellular adhesion molecule-1 (ICAM-1), monocyte chemoattractant protein-1 (MCP-1), tumor necrosis factor-α (TNF-α), and plasminogen activator inhibitor-1 (PAI-1) are potent biological markers for the development of inflammatory and metabolic diseases which may include PCOS [6–8].

ICAM-1 belongs to the super immunoglobulin family and is produced mainly on cell surfaces of endothelial cells, smooth muscle cells, macrophages and activated lymphocytes. It is required for the adhesion of freely moving leukocytes to blood vessel walls and trans-endothelial migration to the vascular intima. This action is crucial in the evolution of atheroma from early to advanced stages. Cleavage of membrane-bound ICAM-1 results in circulating soluble ICAM-1 (sICAM-1) [9, 10]. In prospective epidemiologic investigations, its levels in serum have been linked to abdominal obesity, insulin resistance and cardiovascular disease (CVD) [11].

TNF-α, a cytokine released by adipose tissue, instigates insulin resistance. In the endothelium, the TNF-α signaling pathway, which is regulated by nuclear factor kappa B (NF-KB), governs the expression of adhesion molecules such as sICAM-1 [12]. Many studies have found that women with PCOS have significantly higher ICAM-1, MCP-1 and TNF-α than women without PCOS [13, 14].

Furthermore, MCP-1 is a chemokine that induces insulin resistance in adipocytes and skeletal muscle cells and recruits monocytes and T lymphocytes to inflammatory sites. MCP-1 is a well-known chemokine involved in macrophage recruitment. As a result, targeting MCP-1 may help reduce macrophage-induced inflammatory effects on adipose tissue [15].

Lastly, the critical endogenous regulator of fibrinolytic systems is PAI-1, the coagulation marker. PAI-1 controls fibrinolysis activity, promoting insulin-resistant conditions such as central obesity, metabolic diseases, non-insulin-dependent diabetes and thrombosis progression [16]. Several investigations have found that PCOS women had increased plasma PAI-1 [17, 18].

For PCOS women who do not desire fertility OCPs are the conventional first choice of therapy apart from initial weight reduction and lifestyle modifications. They are well recognized clinically as an efficient tool for regularizing cycles and androgen level amelioration [19]. However, OCPs are associated with venous thrombosis, stroke and derangement of several metabolic parameters in the general population [20, 21]. Earlier, we reported on the repercussions of OCPs on the concentration of metabolic, coagulation and inflammatory markers in serum among women with PCOS [22–25]. However, there is a lack of literature at the molecular level on the various effects of OCPs on women with PCOS. Hence, we conducted this research to compare the ICAM-1, TNF-α, MCP-1 and PAI-1 mRNA profiles between drug-naive PCOS women versus those women with PCOS who obtained OCPs as treatment.

## Material and methods

### Subjects

This cross-sectional survey was conducted at the Department of Endocrinology, Sher-i-Kashmir Institute of Medical Sciences (SKIMS), from April 2015 to March 2018. The Institutional ethics committee approved the study under IEC No: SIMS 131/IEC-SKIMS/2013-6479: dated 09-07-2013.Women were required to fulfill Rotterdam 2003 criteria [26] for the diagnosis of PCOS. Women diagnosed with PCOS who volunteered to participate were required to complete an informed consent form to be enrolled in the study.

### Sample size

Statistical tools Epi Info 7 and Software G power 3.1.5 were used to calculate the sample size; the power (1-beta) = 80% was calculated with a 95% confidence level. 25 subjects in the drug-naive group and 25 subjects in the OCP group were deemed sufficient based on calculations.

### Inclusion criteria

Two groups of patients were included:

1. *Drug-naive group: (N = 25)*

PCOS women who were freshly diagnosed with PCOS and had not received any medication for the treatment of PCOS or any other medicine known to interfere with metabolic, coagulatory, or inflammatory parameters for at least six months before being enrolled in the study.

2. *OCP-treated PCOS patients: (N = 25)*

Newly diagnosed PCOS women already on OCPs (Ethinyl estradiol-0.03 mg and levonorgestrel-0.15 mg) for ≥ 6 months were collected from gynecology clinics.

### Exclusion criteria

Women with diabetes or hypertension were eliminated from the study. Secondary causes of hyperandrogenism, such as 21-hydroxylase deficiency, Cushing's syndrome, hypothyroidism, hyperprolactinemia, and androgen-secreting tumors, were also eliminated by appropriate clinical and laboratory tests. Women who had received any medication for the treatment of PCOS or any other medicine known to interfere with metabolic, coagulatory, or inflammatory parameters at the time of enrolment in the study were again debarred from being part of the study.

### Study design

To avoid heterogeneity, in our study, we selected only one type of OCP with a fixed composition (Ethinyl estradiol-0.03 mg and levonorgestrel-0.15 mg). This particular combination was chosen because this is the most standard type. Knowing the deleterious effect of OCPs on the general population, OCPs were not prescribed from our side; instead, such patients were collected from gynaecology clinics only. Also, baseline data of each OCP-treated PCOS woman was obtained from the respective gynaecology clinics.

### Clinical assessment

A comprehensive proforma was completed by participants from both groups, recording medical details of the history of their menstrual cycles, weight, height, acne vulgaris and excessive hair growth. Oligomenorrhea was identified as menstrual cycles decreased to 8 cycles in twelve months or a gap of greater than 35 days between two cycles, while amenorrhea was identified as menstrual cycle discontinuation for six months. We performed a hirsutism evaluation using the updated Ferriman–Gallwey score (FG score) [27] by counting the density and growth of hair on nine defined body sites. A score of greater than 8 indicates considerable hair growth. We collected relevant clinical details and performed relevant laboratory tests according to the respective requirements to rule out secondary causes of hyperandrogenism.

### Laboratory evaluation

After 10–12 h of fasting, 5 ml of venous blood was drawn from the patient to investigate glucose, insulin, hormones [thyroxine (T4), thyroid stimulating hormone (TSH), luteinizing hormone (LH), follicle-stimulating hormone (FSH), prolactin (PRL), cortisol, 17 hydroxy progesterone (17OHP), testosterone], lipid profile, liver and kidney function. An oral glucose tolerance test (OGTT) was done with 75 g of glucose dissolved in 300 ml of water, followed by sampling after 60 min and 120 min of the glucose load.

Hormone level investigation included 17-OHP for excluding congenital adrenal hyperplasia, TSH and T4 to exclude hypothyroidism, PRL to exclude prolactinoma, cortisol to exclude Cushing's syndrome, testosterone to predict hyperandrogenism and excluded adrenal androgen secretion and gonadotropins i.e. LH, FSH to rule out gonadal dysfunction.

### Assays

The radioimmune assay (RIA) technique was used to assess the parameters like testosterone, T4, Cortisol and 17 hydroxyprogesterone (17OHP). TSH, LH, FSH and PRL were estimated with immunoradiometric assay (IRMA) in duplicate using commercially available kits. The Diagnostic Product Corporation (DPC) USA kit for LH, PRL and FSH. DIASORIN, northwestern Ave for T4 and cortisol. IMMUNOTECH, France, for testosterone, TSH and 17-OHP.The glucose oxidase peroxidase (GOD-POD) (URILAB; India) method was used for glucose (mg/dl) estimation. Cholesterol was estimated using the CHOD-PAP method (DIAB; Austria). Triglyceride levels were calculated using the GPO-PAP method (DIAB; Austria). High-density lipoprotein (HDL) cholesterol and low-density lipoprotein (LDL) cholesterol were tested on Hitachi 912, Japan using the new method of clearance (RANDOX; UK). Plasma insulin was measured by electrochemiluminescence [28] (Cobas e411, Roche Diagnostics Limited, Charles Avenue, West Sussex). Sensitivity, specificity, inter-assay and intra-assay coefficients of variation were within the prescribed limits as per the manufacturer's protocol.

### Calculations

The body mass index (BMI) was derived by dividing the body weight (kg) by body height squared (m2). Insulin sensitivity was assessed employing three parameters: (1) The homeostasis model assessment insulin resistance index (HOMA-IR) (2) The fasting glucose/insulin ratio (FGIR) (3) The quantitative insulin sensitivity check index (QUICKI). HOMA-IR index was calculated using the formula: (Fasting insulin [IU/mL] × fasting glucose [mg/dL]/405. FGIR values were calculated using the formula: Fasting glucose (mg/dL)/fasting insulin (IU/mL). QUICKI values were calculated using the formula: 1/[log fasting insulin (IU/mL) + log fasting glucose (mg/dL)]. Increased HOMA-IR, decreased QUICKI and decreased FGIR scores denote insulin resistance or reduced insulin sensitivity [29, 30].

The concentration of mRNA was assessed directly using the critical threshold (CT) value. Relative quantitation results were obtained using the comparative CT method, also known as the ΔΔC_T_ method. The relative gene expression level was reported using the traditional 2^−ΔΔCt^ method (expressed as relative expression units) [31].

$$\begin{aligned} & {\text{Where}}, \, \;\;\Delta \Delta {\text{C}}_{{\text{T}}} = \Delta {\text{C}}_{{{\text{T }}({\text{sample}})}} - \, \Delta {\text{C}}_{{{\text{T }}({\text{calibrator}})}} \\ & \Delta {\text{C}}_{{{\text{T}}\left( {{\text{sample}}} \right)}} = {\text{C}}_{{{\text{T }}({\text{target gene}})}} - {\text{ C}}_{{{\text{T }}\left( {\text{reference gene}} \right){\text{ sample}}}} \\ & \Delta {\text{C}}_{{{\text{T}}\left( {{\text{calibrator}}} \right) \, }} = {\text{C}}_{{{\text{T }}({\text{target gene}})}} - {\text{ C}}_{{{\text{T }}\left( {\text{reference gene}} \right){\text{ calibrator}}}} \\ \end{aligned}$$

CT reflects the threshold cycle (i.e.), the intersection between an amplification curve and a threshold line. It is a proportional measure of the target concentration in the polymerase chain reaction (PCR) process. The CT values in the sample are inversely proportional to the amount of target nucleic acid. More mRNA in the sample lowers the CT value [32, 33].

## RNA extraction

Approximately 6 ml of venous blood samples from drug-naive PCOS and OCP-treated PCOS women were collected in ethylenediaminetetraacetic acid (EDTA) tubes and diluted in a Diethyl pyrocarbonate (DEPC) treated falcon tube to the same volume of Phosphate buffered saline (PBS). The diluted peripheral blood was poured in a 3:1 ratio onto the ficoll-hypaque and centrifuged at room temperature for 30 min at 500 g without decelerating the speed. The population of mononuclear cells at the ficoll-hypaque interface was collected and transferred into a fresh DEPC-treated falcon tube using a pipette. In the DEPC falcon tube, 1 ml of blood cells were suspended in 4–5 ml of PBS and the contents were centrifuged for 10 min at 500 g. After centrifugation, the supernatant was disposed and the total pellet was obtained. 1 ml of PBS was added to the DEPC-treated Eppendorf tube. The pellet was blended with PBS by gently flicking the tube and centrifuged for 5 min at 500 g.

Total RNA was collected by Trizol reagent (Invitrogen) from the mononuclear cells using the Chomiczyki and Sacchi methods [34]. 1% agarose gel was used to run RNA obtained from samples to check the quality of RNA and the presence of any DNA contamination. DNase treatment to isolated RNA was given using a DNase kit (Sigma, USA) to rule out genomic DNA interference. The RNA content was determined using spectrophotometry.

### Reverse transcription PCR (RT-PCR)/synthesis of complementary DNA (cDNA)

ThermoScientific Revert Aid First Strand cDNA Synthesis Kit containing oligo DT primers were used for preparing the complementary DNA (cDNA). 1 μg of total RNA, 1 μl oligo (dT) primer and 12 μl RNase-free water reaction were used. The reaction was incubated at 65 °C for 10 min. After that, was added 4 μl of 5 × reaction buffer, 2 μl of 10 mM dNTP Mix, 1 μl of Ribolock RNase inhibitor, 1 μl of RevertAid M MulVRT total volume was made up to 10 μl followed by incubation at 42 °C for 1 h, the reaction mixture was then incubated for 5 min at 70 °C. The cDNA prepared was stored at − 80 °C until real-time PCR was performed. The primers and annealing temperatures of ICAM-1, TNF-α, MCP-1, PAI-1 and βeta-actin (β-actin) used in the current study were constructed using PRIMER 3 plus software and are given in Table 1.

**Table 1:** Primer sequences and annealing temperatures of ICAM-1, TNF-α, MCP-1, PAI-1 and β-actin

Target	Primer sequence (5′ → 3′)	T_m_ °C
ICAM-1	Forward primer TATGGCAACGACTCCTTCT Reverse primer CAT TCAGCGTCACCT TGG	56
TNF-α	Forward primer CCCAGGCAGTCAGATCATCTTC Reverse primer AGCTGCCCCTCAGCTTGA	60
MCP-1	Forward primer GTCACCTGCTGCTATAACTTC Reverse primer TGCTGCTGGTGATTCTTCTA	56
PAI-1	Forward primer AGCTCATCAGCCACTGGAAAG Reverse primer GGAGGACTTGGGCAGAAC CA	64
β-actin	Forward primer TTCCTTCCTGGGCATGGAGT Reverse primer CTGTGTTGGCGTACAGGTCT	60

*ICAM-1* Intercellular adhesion molecule-1, *TNF-α* Tumor necrosis factor-α, *MCP-1* Monocyte chemoattractant protein-1, *PAI-1* Plasminogen activator inhibitor-1, *β-actin* Beta actin

### Real-time PCR (qPCR)

The SYBR Green I procedure was used to amplify β actin and target genes and the amplified product was detected. The qPCR master mix with a buffer, DNA polymerase, deoxynucleoside triphosphate (dNTPs) and SYBR Green I dye was used, as shown in Table 2. Gene expression data were normalized against β-actin. The Taq polymerase, cDNA, or primers were not used for negative control reactions for every PCR experiment.

**Table 2:** qPCR reaction mixture used for amplification of ICAM-1, TNF-α, MCP-1, PAI-1 and β-actin Gene

Reagent	Concentration	Volume
Nuclease free water		8.9 µl
Forward primer	10 pmol/µl	0.3 µl
Reverse primer	10 pmol/µl	0.3 µl
cDNA sample	70 ng/µl	0.5 µl
SYBR Green(MM)	2 X	10 µl
Total volume		20 µl

### Statistical analysis

The statistical interpretation was conducted using SPSS version 20.0(SPSS, Inc, Chicago, IL). Data obtained was expressed as Mean ± SD. An unpaired t-test was used to compare continuous variables between the two groups. Regression analysis was performed to determine variables predicting the expression of inflammatory and coagulatory markers. A probability (p) value less than 0.05 was considered statistically significant. GraphPad Prism 5(GraphPad Software, La Jolla, CA) was used to plot graphs. Statistical software stata 16.0 was used to construct box and whisker plots. Statistical tools Epi Info 7 and Software G power 3.1.5 were used to calculate the sample size.

## Results

A total of 25 PCOS women who received OCPs for six months (cases) and 25 drug-naive PCOS women (controls) were registered. Table 3 summarizes baseline characteristics of three groups (i.e.) OCP group pre-treatment, OCP group posttreatment and drug-naive PCOS group. The mean value of the age of the three groups was comparable 24.8 ± 1.20 years in the pre-treatment OCP group versus 24.8 ± 1.20 in the post-treatment OCP group versus 24.25 ± 1.48 years in the drug-naive PCOS group; p = 0.23. The mean BMI of the three groups was also comparable 23.7 ± 1.12 in the pre-treatment OCP group versus 24.70 ± 1.66 in the post-treatment OCP group versus 23.9 ± 2.21 in the drug-naive PCOS group; p = 0.101. FG score, No. of cycles per year, waist-hip ratio, systolic blood pressure (SBP), diastolic blood pressure (DBP), fasting glucose, 1 h glucose, 2 h glucose, total cholesterol, low-density lipoprotein (LDL) cholesterol, high-density lipoprotein (HDL) cholesterol, triglycerides, total testosterone, fasting insulin, 1 h insulin, 2 h insulin, HOMA-IR, QUICKI, FGIR was significantly different between three groups (p =  < 0.0001).

**Table 3:** Comparison of clinical and laboratory characteristics pre-treatment and post treatment in cases (OCP group) versus controls (drug naive group)

Variables	OCP group (Cases)		Drug naive group (Controls)	P value
	Mean ± SD N = 25 Pre treatment	Mean ± SD N = 25 Post treatment	Mean ± SD N = 25	
Age(years)	24.8 ± 1.20	24.8 ± 1.20	24.25 ± 1.48	0.23
FG Score	13.3 ± 4.4	10.26 ± 5.57	15.58 ± 2.99	0.003
No. of cycles per year	7.3 ± 2.2	11.87 ± 2.9	6.8 ± 3.4	0.001
BMI (kg/m^2^)	23.7 ± 1.12	24.70 ± 1.66	23.9 ± 2.21	0.101
Waist Hip Ratio	0.87 ± 0.01	0.89 ± 0.03	0.87 ± 0.02	0.001
SBP (mmHg)	121.87 ± 1.73	129 ± 0.53	121.41 ± 1.72	0.001
DBP (mmHg)	82.07 ± 1.15	85.8 ± 0.83	81.08 ± 1.16	0.001
LH(IU/l)	6.03 ± 1.83	5.8 ± 2.48	6.05 ± 1.63	0.887
FSH(IU/l)	4.56 ± 2.81	5.13 ± 2.28	4.12 ± 2.31	0.357
Testosterone(ng/dl)	75.61 ± 3.2	44.4 ± 5.99	79.66 ± 4.20	0.001
Blood glucose fasting (mg/dl)	84.21 ± 5.22	89.90 ± 10.5	86.25 ± 5.72	0.03
Blood glucose 1 h (mg/dl)	143.6 ± 1.3	173 ± 1.13	141.6 ± 1.26	0.001
Blood glucose 2 h (mg/dl)	120.6 ± 5.3	145.06 ± 4.44	117.66 ± 4.53	0.001
Insulin fasting (µIU/ml)	13.35 ± 1.2	16.06 ± 4.6	12.75 ± 1.35	0.002
Insulin 1 h (µIU/ml)	53.02 ± 2.2	65.6 ± 2.04	55.08 ± 3.43	0.001
Insulin 2 h (µIU/ml)	52.60 ± 3.89	60.53 ± 13.61	51.58 ± 3.89	0.005
HOMA -IR	2.77 ± 1.02	3.56 ± 1.18	2.71 ± 0.17	0.001
QUICKI	0.34 ± 0.01	0.32 ± 0.02	0.34 ± 0.01	0.001
FGIR	6.30 ± 0.55	5.59 ± 1.02	6.76 ± 0.61	0.001
Cholesterol(mg/dl)	152.25 ± 7.92	194.74 ± 7.25	157.25 ± 8.97	0.001
Triglycerides (mg/dl)	97.36 ± 4.83	148 ± 5.2	96.66 ± 5.89	0.001
HDL-Cholesterol(mg/dl)	47.36 ± 5.3	53 ± 6.59	47.06 ± 4.30	0.002
LDL-Cholesterol(mg/dl)	97.66 ± 5.53	110 ± 12.04	96.66 ± 5.89	0.001

*BMI* Body Mass Index, *FGIR* fasting glucose insulin ratio, *FG Score* Ferrimen Gallwey score, *FSH* follicle stimulating hormone, *HDL* High density lipoprotein, *HOMA-IR* Homeostasis Model Assessment Insulin resistance index, *LDL* Low density Lipoprotein, *LH* luteinizing hormone, *OCP* Oral contraceptive pills, *QUICKI* quantitative insulin sensitivity index, *SD* Standard Deviation

**Fig. 1:**
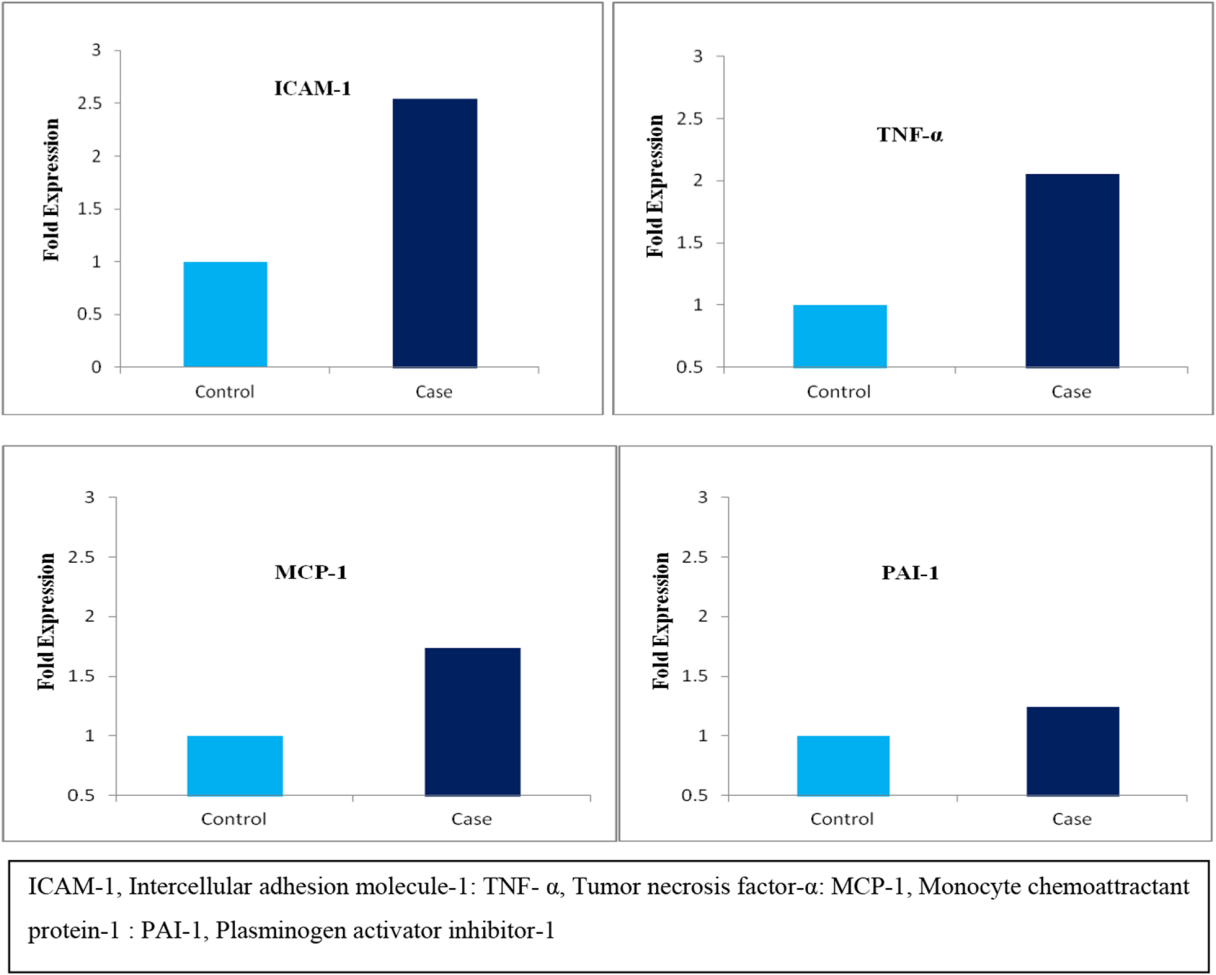
Graphical representation of mRNA fold expression of different genes in drug naive PCOS patients (controls) and OCP treated PCOS patients (cases)

**Fig. 2:**
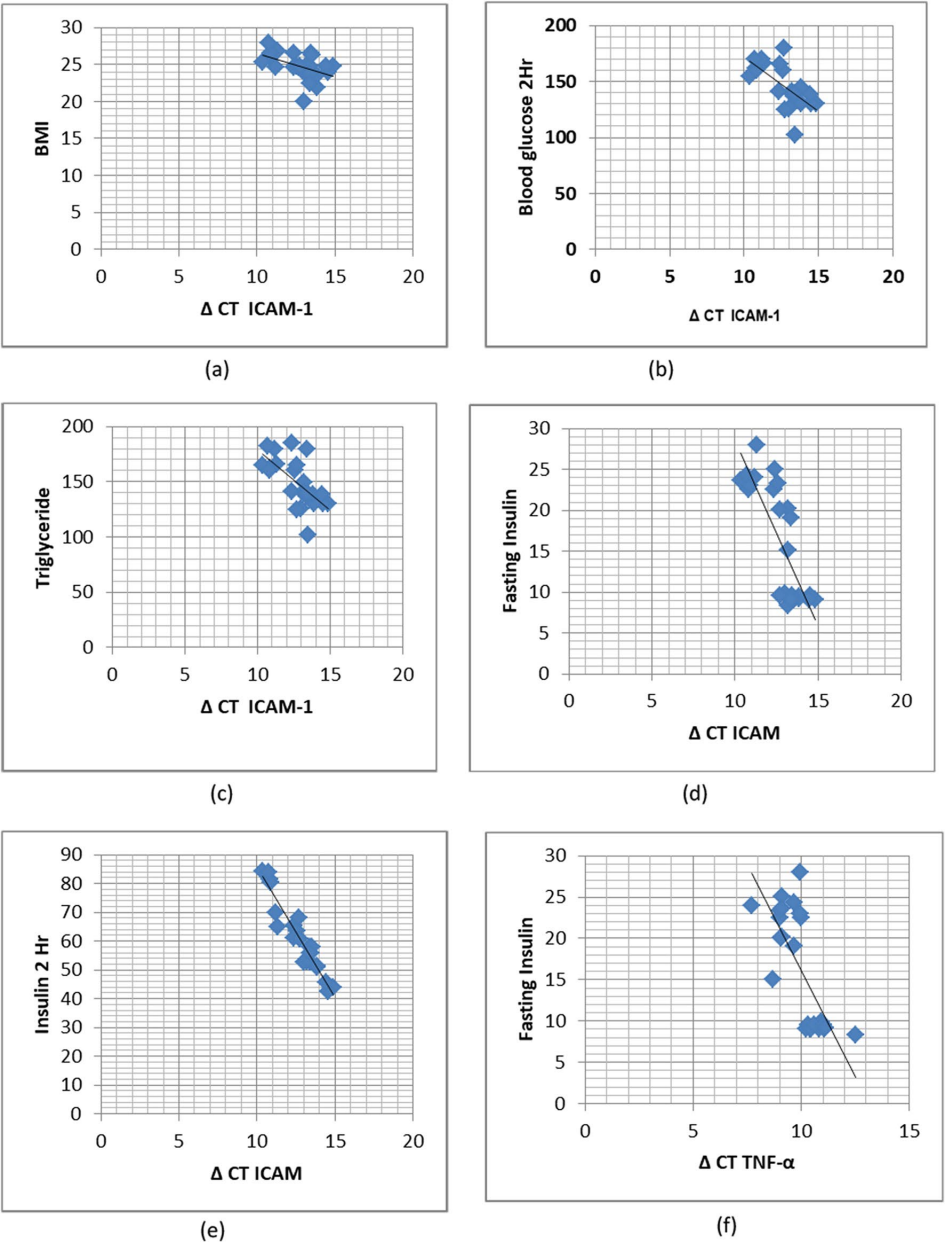

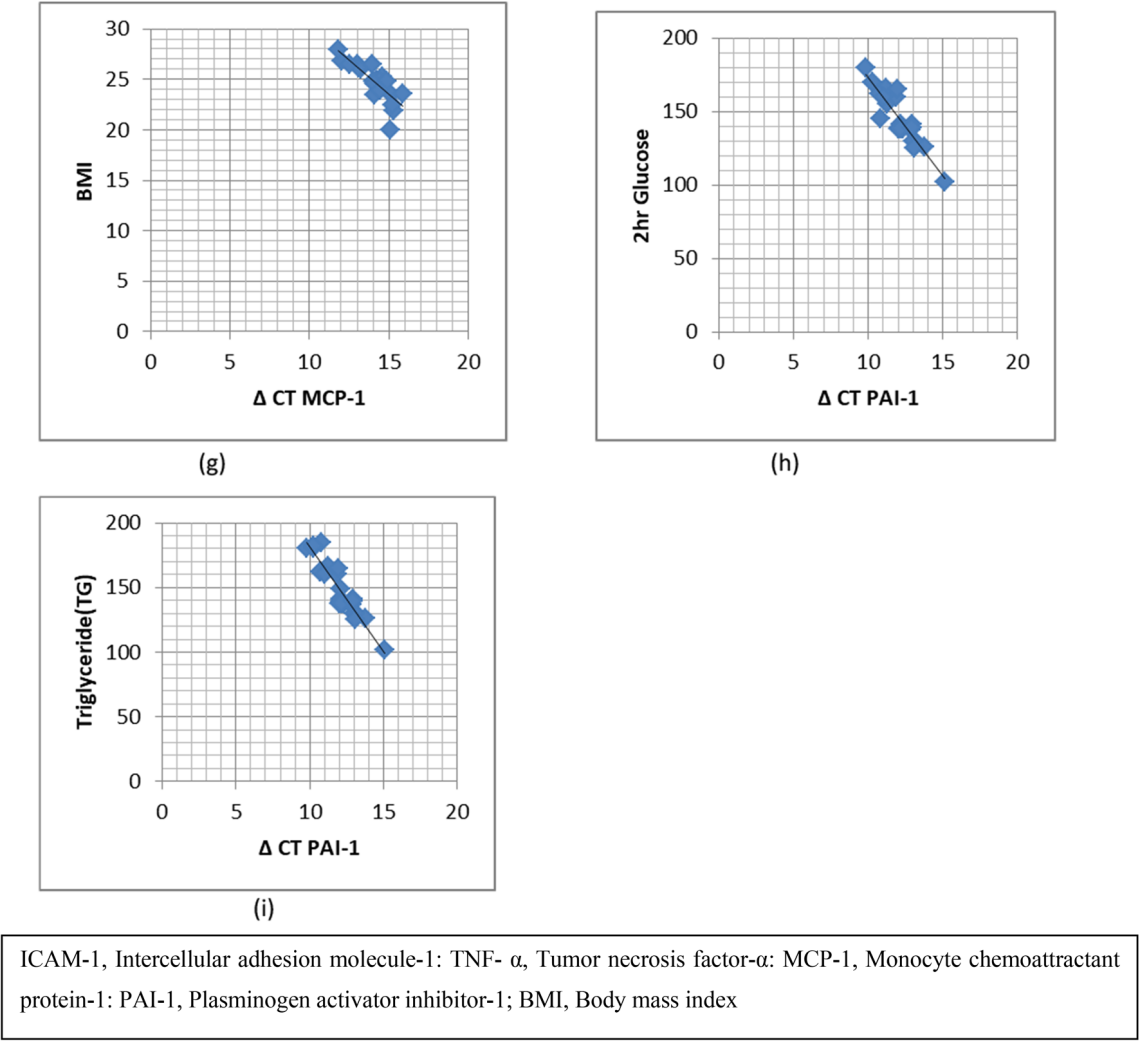
**a** Graph showing correlation between ΔCT ICAM-1 and Body mass index (BMI). **b** Graph showing correlation between ΔCT ICAM-1 and Blood glucose 2 h. **c** Graph showing correlation between ΔCT ICAM-1 and Triglycerides. **d** Graph showing correlation between ΔCT ICAM-1 and Fasting Insulin. **e** Graph showing correlation between ΔCT ICAM-1 and Insulin 2 h. **f** Graph showing correlation between ΔCT TNF-α and Fasting Insulin. **g** Graph showing correlation between ΔCT MCP-1 and body mass index (BMI). **h** Graph showing correlation between ΔCT PAI-1 and Triglyceride. **i** Graph showing correlation between ΔCT PAI-1 and 2 h Glucose. **The ΔCT values are inversely proportional to the amount of mRNA in the sample (i.e.) lower the CT value greater is the mRNA fold expression

Statistically non significant (p > 0.05);

(CT ± SD) for ICAM-1 gene in the drug-naive group (controls) was (30.59 ± 0.72) and in post-OCP treated PCOS group (cases) was (29.53 ± 0.86) (p =  < 0.0001). (ΔCT ± SD) for the drug-naive group (controls) was (14.12 ± 1.08) and for the post-OCP treated PCOS group (cases) was (12.77 ± 1.25) (p = 0.0002). –ΔΔCT was calculated to be 1.35 ± 0.55. In post-OCP treated PCOS patients, the mRNA expression of ICAM-1 increased to 2.54 fold relative to drug-naive PCOS patients (Fig. 1, Table 4). Further, mRNA expression of the ICAM-1 gene in terms of CT values positively correlated with BMI (p =  < 0.0001), triglycerides (p =  < 0.0001), 2 h blood glucose (p = 0.0005), fasting insulin (p =  < 0.0001) and 2 h insulin (p = 0.02) (Fig. 2, Table 5).

**Table 4:** Fold change expression of ICAM-1, TNF-, MCP-1, and PAI-1 gene calculated by ΔΔC_T_ method

Gene	Control (N = 25) CT	Case (N = 25) CT	P value	Control (N = 25) ΔCT	Cases (N = 25) ΔCT	P value	− ΔΔCT	Fold expression
ICAM-1	30.59 ± 0.72	29.53 ± 0.86	< 0.0001	14.12 ± 1.08	12.77 ± 1.25	0.0002	1.35 ± 0.55	2.54
TNF-α	25.92 ± 0.5	24.88 ± 0.7	< 0.0001	11.05 ± 1.08	10.01 ± 1.02	0.001	1.04 ± 0.97	2.05
MCP-1	29.81 ± 0.73	29.01 ± 0.61	0.0001	14.94 ± 1.07	14.14 ± 1.01	0.009	0.8 ± 0.65	1.74
PAI-1	27.28 ± 0.66	26.96 ± 0.78	0.12	12.41 ± 1.43	12.09 ± 1.22	0.39	0.32 ± 0.87	1.24

*ICAM-1* Intercellular adhesion molecule-1, *TNF-α* Tumor necrosis factor-α, *MCP-1* Monocyte chemoattractant protein-1, *PAI-1* Plasminogen activator inhibitor-1, *C*_*T*_ Cycle threshold

Values are presented as mean ± SE

Values having different superscript differ significantly (P < 0.05)

ΔC_T_ = {Mean C_T target_ – Mean C_T reference_}

ΔΔC_T_ = {ΔC_T sample_- ΔC_T calibrator_}

**Table 5:** Association of ICAM-1 mRNA expression with metabolic indices in cases (post OCP treated PCOS group)

Gene	**BMI Kg/m**^**2**^	**Mean ΔC**_**T**_** ± SD**	**Number**	P value
ICAM-1	≥ 25	11.75 ± 1.01	15	< 0.0001**
	< 25	13.79 ± 0.86	10	
	**Blood glucose 2 h mg/dl**			
	≥ 140	12.03 ± 1.03	16	0.0005**
	< 140	13.51 ± 0.48	9	
	**Triglycerides** **mg/dl**			
	< 150	13.81 ± 0.62	8	< 0.0001**
	≥ 150	11.73 ± 1.13	17	
	**Fasting Insulin (µIU/ml)**			
	≥ 10	11.64 ± 1.17	18	< 0.0001**
	< 10	13.9 ± 0.81	7	
	**2 Hr Insulin (µIU/ml)**			
	< 45	13.21 ± 0.54	9	0.02**
	≥ 45	12.33 ± 1.01	16	

*ICAM-1* Intercellular adhesion molecule-1, *BMI* Body mass index, *C*_*T*_ Cycle threshold, *SD* Standard deviation

(CT ± SD) for the TNF-α gene in the drug-naive group (controls) was (25.92 ± 0.5) and in post-OCP treated PCOS group (cases) was (24.88 ± 0.7) (p =  < 0.0001). (ΔCT ± SD) for the drug-naive group (controls) was (11.05 ± 1.08) and post OCP treated PCOS group (cases) was (10.01 ± 1.02) (p = 0.001). –ΔΔCT was calculated to be 1.04 ± 0.97. mRNA expression of TNF-α increased to 2.05 fold in the OCP group relative to drug-naive PCOS patients (Fig. 1, Table 4). Additionally, mRNA expression of TNF-α interms of CT values positively correlated with fasting insulin (p = 0.0007) (Fig. 2, Table 6).

**Table 6:** Association of TNF-α mRNA expression with metabolic indices in cases (post OCP treated OCP group)

Gene	Fasting insulin	Mean ΔC_T_ ± SD	Number	P value
TNF-α	≥ 10	9.3 ± 1.07	16	0.0007
	< 10	10.9 ± 0.81	9	

*TNF-α* Tumor necrosis factor-α, *C*_*T*_ Cycle threshold, *SD* Standard deviation

(CT ± SD) for the MCP-1 gene in the drug-naive group (controls) was (29.81 ± 0.73) and in post-OCP treated PCOS group (cases) was (29.01 ± 0.61) (p = 0.0001). (ΔCT ± SD) for the drug-naive group (controls) was (14.94 ± 1.07) and for post OCP treated PCOS group (cases) was (14.14 ± 1.01) (p = 0.009). –ΔΔCT was calculated to be 0.8 ± 0.65. mRNA expression of MCP-1 significantly increased to 1.74 folds compared with the drug-naive PCOS category (Fig. 1, Table 4). mRNA expression of the MCP-1 gene in terms of CT values positively correlated with BMI (P = 0.002) (Fig. 2, Table 7).

**Table 7:** Association of MCP-1 mRNA expression with metabolic indices in cases (post OCP treated PCOS group)

Gene	BMI Kg/m^2^	Mean ΔC_T_ ± SD	Number	P value
MCP-1	≥ 25	13.49 ± 1.01	13	0.002
	< 25	14.79 ± 0.86	12	

*MCP-1* Monocyte chemoattractant protein-1, *BMI* Body mass index, *C*_*T*_ Cycle threshold, *SD* Standard deviation

(CT ± SD) for PAI-1 gene in the drug-naive group (controls) was (27.28 ± 0.66) and in post OCP treated PCOS group (cases) was (26.96 ± 0.78) (p = 0.12). (ΔCT ± SD) for the drug-naive group (controls) was (12.41 ± 1.43) and for the post-OCP treated PCOS group (cases) was (12.09 ± 1.22) (p = 0.39). –ΔΔCT was calculated to be 0.32 ± 0.87. mRNA expression of PAI-1 was 1.24 fold which was not significantly higher than the drug-naive PCOS group (Fig. 1, Table 4).PAI-1 expression in the OCP group in terms of CT values positively correlated with post-glucose load 2 h blood glucose (p = 0.008) and triglyceride levels (p = 0.001) (Fig. 2, Table 8).

**Table 8:** Association of PAI-1 mRNA expression with metabolic indices in cases (post OCP treated OCP group)

Gene	Glucose 2 h	Mean ΔC_T_ ± SD	Number	P value
PAI-1	≥ 140	11.64 ± 0.9	15	0.008
	< 140	12.54 ± 0.48	10	
	**Triglycerides**			0.001
	≥ 150	11.47 ± 0.88	17	
	< 150	12.71 ± 0.62	8	

*PAI-1* Plasminogen activator inhibitor-1, *C*_*T*_ Cycle threshold, *SD* Standard deviation

**Fig. 3:**
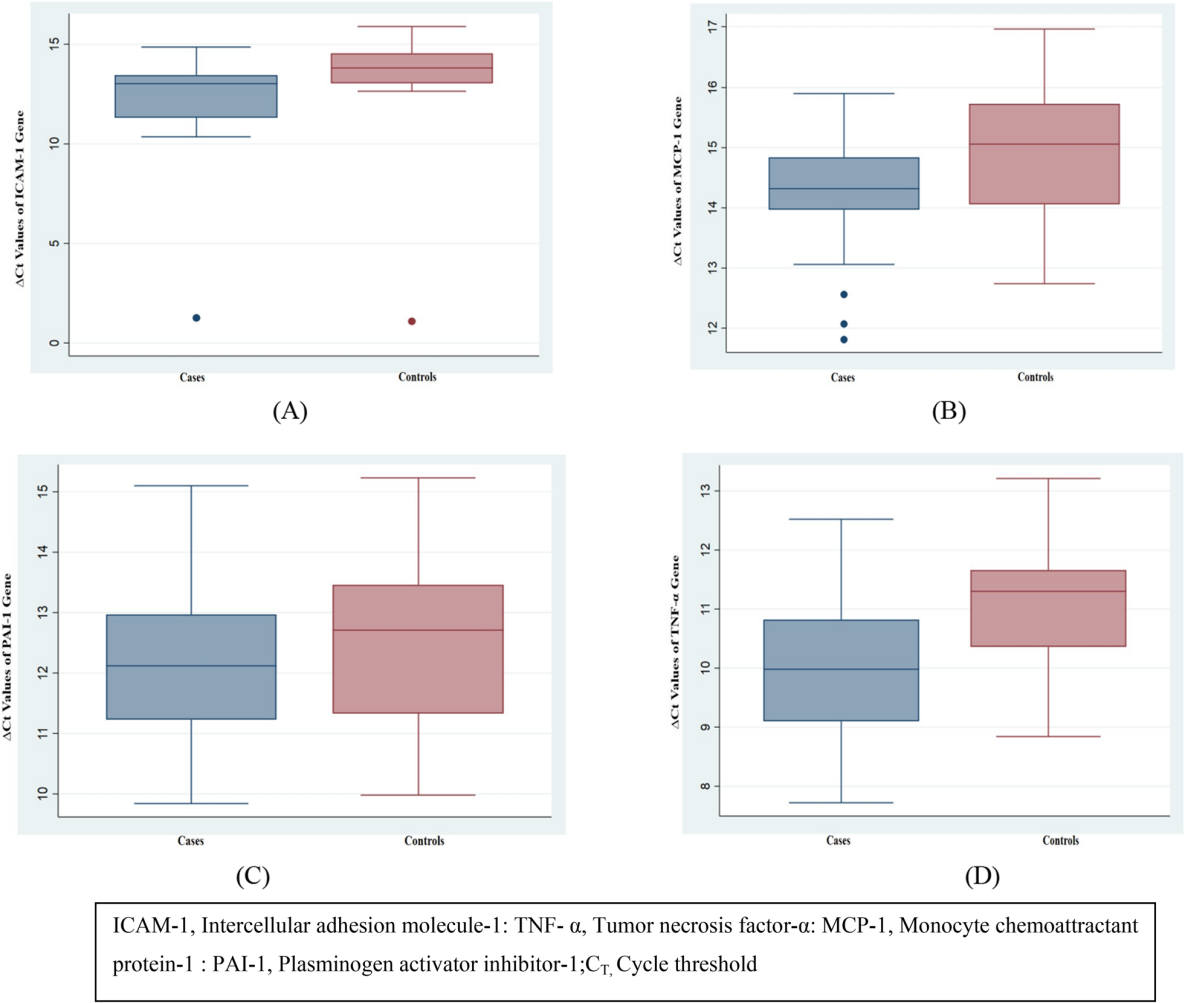
Messenger RNA (mRNA) expression of ICAM-1 (**A**), MCP-1 (**B**), PAI-1 (**C**),TNF-α (**D**) genes as determined by quantitative real‐time polymerase chain reaction in cases and control samples (n = 25). Box and whisker plots describe the relative expression of these genes in cases verses controls. The experiments were performed in triplicates. ΔCt values were determined for all samples by Ct genes‐Ct Beta actin. The bottom and the top of the box represent the 25th and the 75th percentile, respectively, and the band near the middle of the box is the 50th percentile (the median). The end of whiskers represents the smallest and largest observation

Box and whisker plots describe the relative expression of these genes in terms of ΔCt values in cases versus controls are shown in Fig. 3.

On performing regression analysis between ΔCT values (which are inversely proportional to the amount of mRNA in the sample) of selected markers and other clinical and laboratory parameters in the post-OCP treated PCOS group, it was observed that fasting insulin (β = − 0.298; p = 0.025) and 2 h insulin (β = − 0.849; p = 0.00) independently predicted ICAM-1 expression. Fasting Insulin independently predicted the TNF-α expression (β = − 0.745; p = 0.00). BMI independently predicted the MCP-1 expression (β = − 0.798; p = 0.000). Triglycerides independently predicted the PAI-1 expression (β = − 0.798; p = 0.000).

## Discussion

Clinical trials based on biomarkers have proved extremely useful in assessing the efficacy of conventional treatment for any disease. To our knowledge, this is the first study of its kind inquiring into the implications that administration of OCPs (Ethinyl estradiol-0.03 mg and levonorgestrel-0.15 mg) might exert on the mRNA expression profile of inflammatory and coagulatory genes in PCOS women. This study may be of significant clinical importance in treating women with PCOS.

Our findings are consistent with the unfavorable impact of OCPs on the cardiovascular risk profile in the general population, which occurs from the liver's aid in producing procoagulant indicators and the induction of resistance to activated protein C [35]. In our results, in addition to the considerable changes in metabolic indices, we observed ICAM-1, TNF-α and MCP-1 mRNA expression in OCP-treated PCOS women was increased to 2.54 fold, 2.05 fold and 1.74 fold, respectively, compared to drug-naive PCOS women. Our results revealed that the expression of ICAM-1 mRNA was highest, followed by TNF-α mRNA, MCP-1 mRNA and PAI-1 mRNA. A slight increase in fold expression (i.e.) 1.24 folds was observed in the case of PAI-1 mRNA, which could not achieve statistical significance. Further, we looked at the relationship between mRNA expression and various clinical and laboratory indices in OCP-treated PCOS women.ICAM-1 mRNA expression positively correlated with BMI, fasting insulin, insulin 2 h, blood glucose 2 h and triglycerides. TNF-α mRNA expression positively correlated with fasting insulin levels. MCP-1 mRNA expression positively correlated with BMI. PAI-1 expression positively correlated to 2 h blood glucose and triglyceride levels.

We can only compare our findings to studies that are indirectly related to our study because studies similar to ours are unavailable. Several studies have assessed the circulating cytokine levels in PCOS women. In the PCOS population, there are well-known significantly increased levels of ICAM-1 [6, 7], MCP-1 [36, 37], TNF-α [13, 14] and PAI-1 [17, 18] compared to the control group, which represents the early signs of macrovascular problems in these women. One of our recent studies reported that PCOS women have a lower antiinflammatory profile interleukin-10(IL-10) and adiponectin and non-enzymatic antioxidant characteristics than healthy participants [38, 39].

Innumerable studies favor considering OCPs as conventional treatment in PCOS women indicating that OCP administration causes a decrease in free androgens, resulting in decreased new hair development and the growth of terminal hair [40]. OCPs are also responsible for diminishing inflammatory acne count by 30–60%, with 50–90% of patients improving [41]. The estrogen component of OCPs has been demonstrated to subdue LH secretion and diminish ovarian androgen production while simultaneously increasing sex hormone binding globulin (SHBG), which helps to lower free testosterone levels [42]. There is a wealth of information in the literature about the effects of OCPs on glucose tolerance and insulin sensitivity in women with PCOS, indicating that OCPs have a negative impact on these parameters, which can lead to an increased long-term risk of metabolic disorders such as type 2 diabetes mellitus (T2DM), IR and CVD [43–46].

OCPs can promote gene transcription, which boosts mRNA expression. Estrogen enters the cytoplasm after crossing the cell membrane, interacting with estrogen receptors and forming the complex. The complex enters the nucleus and binds to estrogen response elements there. The transcription of inflammatory response proteins is turned on due to this interaction. By interacting with other transcription factors via protein–protein interactions, estrogen connected to estrogen receptors can affect overall gene expression [47–49]. Thus, it is likely that a combination of all of these estrogen-induced intercellular reactions, as well as the form of progesterone in OCP, influences gene expression and increases the development of inflammatory proteins, which in the case of our study is seen as an increased ICAM-1, TNF-α and MCP-1fold expression after treatment with OCP's, despite the lack of much larger fold expression.

We previously assessed the effect of OCP treatment on serum and plasma levels of various proinflammatory and procoagulant markers, including ICAM-1, TNF-α, MCP-1, and PAI-1. Six months of OCP treatment significantly increased serum ICAM-1, MCP-1 and TNF- α levels in women with PCOS [22], but no significant difference was observed in plasma PAI-1 levels [23]. These findings are in line with our current investigation, which looked at the influence of OCPs on the mRNA levels of the same indicators. The impact of OCPs on inflammatory cytokine levels in the serum has been explored in several studies and the results have been mixed. Tatsumi et al. reported that OCP use raised serum ICAM-1 level, which is entirely consistent with the findings of our studies [22, 50]. It has been documented that treatment with OCPs (Ethinyl estradiol and levonorgestrel/desogestrel) elevated inflammatory C-reactive protein (CRP) levels in PCOS women [51]. Contrary to our findings Hemelaar M et al., reported healthy postmenopausal women taking OCPs comprising ethinyl estradiol and gestodene experienced decreased plasma levels of adhesion molecules [52]. In the PCOS women taking OCPs containing ethinyl estradiol and cyproterone acetate, Bilgir O et al. observed the post-treatment values of sVCAM, sICAM, and sE-selectin did not change significantly from the pre-treatment values [53] which, once more, is not consistent with our findings; nevertheless, the OCPs utilized in both investigations had a different composition from what we did in our study.

On the other hand, another study found that treating PCOS patients with OCPs containing cyproterone acetate decreased high-sensitivity C reactive protein (hsCRP) and follistatin levels. Still, the OCPs used in the two trials differed [54]. In addition, we have previously reported that OCPs have an unfavorable impact on metabolic indices, plasma and serum levels of coagulatory and inflammatory markers of PCOS patients, which was strongly linked to the development of T2DM, dyslipidemia, coronary artery disease and thrombotic events [24, 25]. Furthermore, our findings were consistent with those of Luque-Ramirez et al. They found that OCPs negatively affected blood clotting tests in PCOS women compared to metformin, implying that OCP use increased the risk of venous thromboembolism in PCOS women [55].

Inflammatory cytokine mRNA levels in women with PCOS have only been studied in a few research studies that potentially provides indirect evidence for our findings. Vascular cell adhesion molecule (VCAM-1) and ICAM-1 mRNA fold expression were considerably higher in PCOS patients than in healthy controls in a recent study. However, resistin, interleukin-6 (IL-6), TNF-α and MCP-1 mRNA fold expression were not different between the two groups [56]. In another investigation, no differences in mRNA expression of inflammatory markers were detected between PCOS patients and healthy controls with the same body composition [57]. Few studies have examined the influence of medication like metformin on the mRNA profile of inflammatory genes. In one such study, after treatment with metformin, the expression of vaspin was lowered [58]. In PCOS women, however, metformin therapy increased adipolin mRNA expression [59]. However, no research has been done to date to investigate the influence of OCPs on the mRNA profile of inflammatory genes in PCOS women, limiting the ability to compare and validate our findings.

Although we found a positive relationship between the mRNA expression of inflammatory markers and numerous metabolic parameters in our study, however, while we did not explicitly examine the link between elevated inflammatory mRNA expression and thrombosis formation in this study, we did explore the influence of OCPs on coagulation cascades in PCOS women in our recent investigations [24, 25]. When compared to drug-naive PCOS women, OCP users had significantly higher plasma levels of clotting factors such as tissues factor (TF), factor XIa, tissue plasminogen activator (tPA), factor Va, D-dimer and thrombin antithrombin (TAT) III complex values, as well as statistically significant lower prothrombin time (PT) and activated partial thromboplastin time (APTT) values, implying that the drug's interaction with various metabolic pathways raises the risk of hypercoagulability, venous thromboembolism and thrombotic events [24, 25]. In the current study, we detected an insignificant modest increase in PAI-1 expression in PCOS patients after six months of OCP usage. There hasn't been any study that looked into the effect of OCPs on PAI-1 mRNA expression till now. Nonetheless, just a few research have looked at the impact of OCPs on levels of PAI-1in the serum and the results are more ambiguous than definitive [60–62]. No significant difference was observed in PAI-1 levels of the two groups after six months of treatment with OCPs containing levonorgestrel. In line with our findings, earlier research has suggested that using OCP containing levonorgestrel did not affect PAI-1 levels [61]. Further, Teede HJ et al. reported a substantial decrease in PAI-1 levels in PCOS females treated with OCPs containing cyproterone acetate [62].

In our study, fasting insulin and 2 h insulin independently predicted ICAM-1 expression in the post treatment OCP group. Fasting insulin independently predicted TNF-α expression in the post treatment OCP group, which validates that IR is directly interlinked with various low-grade tissue-specific inflammatory responses induced by multiple proinflammatory mediators, notably proinflammatory cytokines such as ICAM-1 and TNF-α which plays a crucial role in the pathogenesis and development of T2DM [63–65]. Chronic exposure to proinflammatory mediators stimulates the activation of cytokine signalling proteins which ultimately block the activation of insulin signalling receptors in βeta-cells of pancreatic islets [66]. BMI independently predicted expression of MCP-1 expression in post treatment OCP group. Our findings support the idea that increased MCP-1 cause adipocyte dedifferentiation and contribute to diseases connected to obesity and hyperinsulinemia [67]. Triglycerides independently predicted the PAI-1 expression in post treatment OCP group in our study which supports the prior research that PAI-1 contributes mechanically to a number of symptoms of the syndrome such as obesity, hypertension and insulin resistance [68] and that the crucial regulator of hepatic lipid metabolism is the PAI-1 [69].

The small sample size is the limitation of our study. Since the inclusion and exclusion criteria were tight, just one particular arm of OCPs was chosen, and the OCPs were administered for the prescribed time. We had to abide closely by the inclusion and exclusion criteria outlined for the study; otherwise, the number of subjects could have been increased.

Pharmacologic therapy is critical in managing patients with PCOS when lifestyle modifications fail to achieve therapeutic goals; still, short-term use of OCP in women with PCOS suggests worsening of already existing insulin resistance and glucose tolerance abnormalities, although the effect seems mild. The significant fall in serum testosterone by OCP translates into many clinical benefits, such as the regularization of menstrual cycles and improvement in acne and other androgenic features. However, OCP use was associated with elevated coronary artery disease risk factors such as total and LDL cholesterol levels. OCP use can alter the expression of genes involved in inflammatory and coagulatory pathways, which can worsen the inflammatory and coagulatory pathophysiology among PCOS women.

Our findings pave the road for well-designed, large-scale randomized, longitudinal molecular trials with a cohort of PCOS women to create improved PCOS management options in clinical practice that will be of immense public importance.

## Conclusion

We conclude that OCPs may have a harmful impact on the metabolic and proinflammatory profile of PCOS women. As a result, OCP use may be in double jeopardy for women with PCOS because they already have a higher incidence of low-grade inflammation. These results reiterate the need to seek alternative therapeutic targets for PCOS management. Anti-inflammatory drugs that target the inflammatory cycle could be a therapeutic substitute or complement to the current treatment.

## Data Availability

The data used and analyzed during the current study are available from the corresponding author upon reasonable request.
